# Physiological Measures of Acute and Chronic Pain within Different Subject Groups: A Systematic Review

**DOI:** 10.1155/2020/9249465

**Published:** 2020-09-03

**Authors:** H. Korving, P. S. Sterkenburg, E. I. Barakova, L. M. G. Feijs

**Affiliations:** ^1^Department of Behavioral and Movement Sciences, Clinical Child and Family Studies, Amsterdam Public Health, Vrije Universiteit Amsterdam, Van der Boechorststraat 7, Amsterdam 1081 BT, Netherlands; ^2^Department of Industrial Design, Future Everyday Group, Eindhoven University of Technology, Groene Loper 3, Eindhoven 5612 AE, Netherlands; ^3^Bartiméus, Oude Arnhemse Bovenweg 3, Doorn 3941 XM, Netherlands

## Abstract

**Results:**

The methods' heart rate variability and electroencephalogram show clear and consistent results as acute pain assessment. Magnetic resonance imaging can measure chronic pain. Ordered by invasiveness and vulnerability, a trend shows that the invasive methods are used more with less vulnerable subjects. Only instruments used for skin conductance and automatic facial recognition have a lower-than-average technological maturity.

**Conclusions:**

Some pain assessment methods show good and consistent results and have high technological maturity; however, using them as pain assessment for persons with ID is uncommon. Since this addition can ameliorate caregiving, more research of assessment methods should occur.

## 1. Introduction

When a person is unable to communicate that he or she has pain, for example, those with severe or profound intellectual disability (S/PID), this may cause unnoticed suffering. They might receive less pain relief medication or receive it too late, and not knowing whether there is pain creates doubt in caregivers. A verbal person without disabilities can be asked to self-report about their pain, but this is impossible in persons with S/PID. Observations seem like a good alternative, but they have been shown to miss subtle signs of pain specific in adults with S/PID [1]. The assessment of physiological signs of pain can be a good alternative for persons unable to communicate about pain. Furthermore, physiological assessment might be more accurate than observations for this group.

Pain research among persons with intellectual disabilities is scarce [2], and the research focusing on physiological signs of pain in this group even more so. De Knegt et al. [2] conclude that specific behavioral indicators are often and consistently mentioned as indicators for pain in people with intellectual disabilities, but also indicated that there are physiological response clues that can be examined to measure pain. This indicates that physiological assessment of pain can be introduced as an addition in caregiving for persons with S/PID. This provides an incentive for researchers to add physiological assessments to pain research among persons with intellectual disabilities.

While reliability is a very important aspect of instruments used to physiologically assess pain, aspects of instrument invasiveness and patient vulnerability should also be considered. Not all physiological pain-detection instruments are equally (non) invasive, and not all patients are equally able to express and communicate emotions or give consent. For example, when deciding which instrument to use for pain assessment for a comatose patient, a physician and the patient's legal representative might decide to use the less invasive method, until that time that the patient is able to give consent. This stems from the Universal Declaration of Human Rights, which recognizes the “inherent dignity” of every human being [3] and thus the responsibility to consider the invasiveness of methods.

When using a technical instrument to assess physiological signals that can indicate pain, technological maturity is a factor which relates to the instrument's reliability. Technological maturity can be assessed using technology readiness levels (TRLs), as first conceptualized in 1974 by The United States National Aeronautics and Space Administration [4, 5]. TRLs were adopted by the US Department of Defense in their Defense Acquisition Guidebook [6] and are rated on a scale which corresponds to the reported fundamental and practical steps for development and testing of instruments [4]. Using TRLs to assess the maturity of instruments used to detect physiological signals of pain gives additional information to make comparisons between assessment methods and instruments.

Thus, there is a need to use physiological methods to detect pain in subject groups. In this review, the relationship between instrument invasiveness and patient vulnerability will be examined, as well as the technological maturity of most-used pain assessment instruments. The research questions are as follows: (1) which physiological methods to detect pain are used with which subject groups? (2) Which methods with which subject groups indicate that pain assessment is possible and reliable, and are these results consistent among studies? (3) Which pattern concerning physiological assessment of pain can be identified when we compare the invasiveness of the physiological measure and the vulnerability of the subject groups? (4) What are the technology readiness levels for physiological instruments used to measure pain?

## 2. Materials and Methods

### 2.1. Included Articles

This systematic review was conducted following PRISMA-guidelines [7]. In Table 1, the inclusion and exclusion criteria are reported. The included articles had to be reviews or meta-analyses, which described original research articles. These research articles had to describe research using at least one physiological method to detect pain in humans. The pain studied in the research articles could be present already, or it could be induced by either a medical procedure (e.g., a vaccination) or a scientific method (e.g., a deliberate painful experience). Authors were contacted when a full-text of the review was unavailable online. The reviews were published in Dutch, English, French, German, and Spanish between January 2007 and March 2019. This 12-year period was chosen to get a well-rounded image of the use of physiological methods for pain in the late 20^th^ and early 21^st^ century.

**Figure 1:**
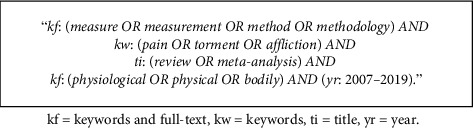
Search terms used in all database searches.

A systematic search of review articles was conducted on WorldCat, Wiley Online Library, SpringerLink, IEEE Publications Database, ScienceDirect, and Cochrane Library between December 1^st^, 2018, and March 31^st^, 2019, via the University LibSearch on Worldcat.org. The search strategy consisted of a combination between free-text and title words, which is shown in Figure 1. This search gave 1.984 results.

Of the 1.984 results, 200 (10.1%) were randomly selected. The first author (HK) and two other researchers (SD and GK) independently coded the title and abstract of the 200 articles on inclusion and exclusion criteria. There was a 100% agreement on the inclusion and exclusion of these 200 articles (Cohen's kappa = 1.00). The first author (HK) then conducted a title and abstract screening of the remaining 1.784 articles. After this screening, 63 reviews were included in the full-text coding.

Of these 63 reviews, 13 (20.6%) were randomly selected. The first author (HK) and another independent researcher (LW) screened the full-text on inclusion and exclusion criteria. Both researchers agreed 100% on which of those articles should be included and which should be disregarded (Cohen's kappa = 1.00). In this phase, reviews were excluded when physiological methods were used, but not to assess pain, or when pain was experimentally induced, but not assessed. After this second phase, 29 reviews remained. A flowchart of the entire search strategy is shown in Figure S1.

### 2.2. Quality Assessment

Of the 29 reviews, a random subset of 6 (20.7%) was assessed on quality by HK and GK. For this quality assessment, the CASP checklist [8] was used. This checklist assesses reviews with ten questions on three aspects: (a) validity of results, (b) precision of results, and (c) usefulness of results. After discussion of results and compromise on when an aspect could be considered adequately conducted, HK assessed the remaining 23 reviews according to the agreement made.

For each question, it was assessed whether this element was adequately conducted (noted by a plus sign (+)) or inadequately carried out (noted by a minus sign (−)). Moderately adequate elements were noted by a slash sign (/), and when it was unclear whether an element was adequately carried out, this was noted by a question mark (?). As the CASP checklist was designed for educational purposes, a scoring system is not suggested. The number of adequately carried out elements was counted, and it was noted whether each review was carried out according to a systematic method. Results of the quality assessments are given in the supplementary material (Table S1).

### 2.3. Literature Taxonomy

A taxonomy was developed using the 540 articles described in the 29 reviews. The taxonomy comprised of the physiological method used to detect pain (modality) for each subject group. The first step in this taxonomy was to list all modalities used and all subject groups included in the reviews. The modalities were then scored on invasiveness and the subject group on vulnerability. The first two authors (HK and PS) and an independent researcher (GK) scored the modalities independently. Scores on invasiveness could vary between 0 and 6 and scores on vulnerability between 0 and 5 (Table 2) and when needed half points were given. Subsequently averages were calculated for each modality and each subject group.

The three researchers (HK, PS, and GK) scored the invasiveness of modalities and the vulnerability of subject groups according to an agreed-upon scoring model (Table 2). Invasiveness was based on two aspects: “how drastic is the method?” which considered both the patient's physical integrity and their privacy on a scale from 0 to 3 and “how long would analysis of results take?” on a scale from 0 to 3. A higher score indicates a more invasive modality. The modalities started at the least invasive “respiratory rate,” which is not at all drastic for the patient and fast, to “conducting genetic research,” which both use bodily fluids and take generally more than 1 day to analyze.

Vulnerability was based on three aspects: “what are the communicative capabilities of the group?” on a scale from 0 to 2; “how competent is the group?” on a scale from 0 to 2; and “does the group have a (medical) condition (including an intellectual disability)?” on a scale from 0 to 1. Higher scores indicate less vulnerability. Half points were given when a modality's physical invasiveness was more than moderate but less than very drastic. Nonverbal (comatose) patients, who have limited nonverbal expressivity, cannot decide their own fate and who have an illness or injury were considered the most vulnerable group, closely followed by people with severe ID. Healthy adults were considered the least vulnerable group. In this way, an average score was calculated for 18 physiological modalities for pain and 7 subject groups on which those modalities were used. The average scores are shown in Table 3.

Most assessment methods are used to measure pain at the moment of measurement. They are monitored during surgical procedures, during vaccinations, or during experimentally induced pain. During these procedures, heart rate, pupillometry, and needle-based EMG can give information about the existence of pain. Other methods, such as (f) MRI, PET, and genetics, are most often used to assess changes in the brain or the genes as a result of long-term chronic pain. An MRI can show the density of different brain regions and is used to search for differences between chronic pain patients and healthy controls. Genetic research can look for inherent changes in genes in chronic pain patients, or for damage in DNA.

A random subset of 6 reviews (20.7%) was made, and HK and another independent researcher (SC) independently assessed the used modality for pain and the subject group on which this modality was used. This assessment was done as follows: if a study assessed pain with both heart rate variability and skin conductance on both healthy adults and verbal patients (e.g., four unique combinations: heart rate variability and healthy adults, heart rate variability and verbal patients, skin conductance and healthy adults, and skin conductance and verbal patients), this led to four unique codes in the taxonomy. If a study utilized three modalities on three subject groups, this was coded as 9 combinations and so on. After both researchers coded the 6 reviews, they held a meeting to discuss terminology. Agreement before the meeting was high (Cohen's kappa = 0.88), and in the meeting, the researchers reached consensus on aspects that were still unclear, such as when an infant is a neonate, and when a patient is comatose. HK assessed the remaining 23 reviews and removed duplicate articles. The assessment resulted in 1.054 unique combinations of article, modality, and target group.

The coded combinations of modality and target group were then visualized in a scatter plot with the subject group on the *y*-axis and modality on the *x*-axis. The modalities and subject groups were ordered according to their averages on invasiveness or vulnerability, according to the model previously described (see also Table 2). The point where the two axes come together is the place of least invasive modality and most vulnerable group, point 0, 0. On this point on the scatter plot, the measurement of pain via respiratory rate connects with (noncommunicative) comatose patients. The point where the most invasive pain assessment method (genetics) meets the least vulnerable subject group (healthy adults) is point 6, 5.

### 2.4. Pain Assessment per Method

For each review, the evidence of pain measurement methods was assessed and results were gathered. The results will be displayed per review further on in this article. Where this is specified in the review, the used instruments to measure pain and the subjects the instrument was used on will also be mentioned.

### 2.5. Technology Readiness Level

**Figure 2:**
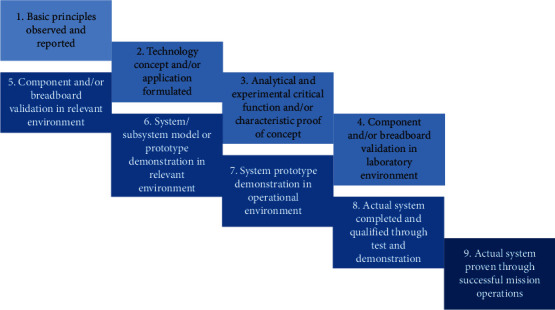
Technology readiness level descriptions used by the United States Department of Defense [6]. The figure is adapted from a figure used by the European Association for Research and Technology Organizations [9] to improve legibility.

All methods to measure pain with physiological signals were then ordered according to their technology readiness level (TRL). The United States Department of Defense has published their version in 2010, which was adapted for this assessment (Figure 2). The translation from each level was done in accordance with the evaluation of technological instruments to physiologically measure pain in a clinical setting and is included in the supplemental material (Figure S2).

For each method of measuring pain physiologically, one technical instrument was chosen to assess its TRL. This instrument represents the most-used technical method to measure that particular physiological signal; for example, skin conductance is more often measured with stick-on electrodes, and less often with wearable electrodes. When more than one method is used frequently or the most-used method could not be determined, a choice was made. With two or more equal methods, based on TRL, a random choice was made between the methods. When the methods differed much on TRL, the most mentioned method in the articles reviewed was chosen.

## 3. Results and Discussion

### 3.1. Quality Assessment

The quality of each of the 29 reviews was assessed with the checklist of the Critical Appraisal Skills Programme [8]. The quality score of the reviews was generally high (median = 7/10). The reviews scored mostly well on their validity and their usefulness but lacked precision in their results. It was not always clear whether the authors took enough steps to ensure that all relevant studies were included in the reviews. Also the generalizability of the articles was sometimes limited, which also limited the generalizability of the reviews. Out of the 29 reviews, only ten (34.5%) mentioned making use of a systematic method for conducting the review and fifteen of the reviews (51.7%) assessed the quality and risk of bias of the articles included.

### 3.2. Literature Taxonomy

**Figure 3:**
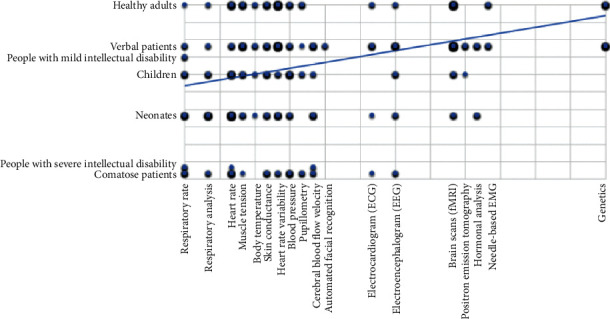
Taxonomy with measurement methods on the *x*-axis, ordered from least to most invasive, and subject groups on the *y*-axis, ordered from most to least vulnerable.

There were 1.054 combinations of modality and subject group, from 540 articles (published between 1972 and 2017) reported in 29 reviews (published between 2007 and 2019). Each review described more than one article on physiological measures for pain. Only articles where the physiological modality and the subject group were clearly described were used to create the taxonomy. The modalities are ordered on the *x*-axis according to invasiveness (with the more invasive measures further to the right) and the subject groups on the *y*-axis according to vulnerability (with the least vulnerable groups further to the top). The taxonomy shows that the more invasive modalities are more often used on the least vulnerable subjects, while less invasive measures are used on subject groups of all vulnerability levels (Figure 3). The lack of studies that looked at physiological measures for pain in subject groups with mild or severe intellectual disability can also be seen in the taxonomy. This is not an effect of studies on pain. There is very little research done on subjects with intellectual disabilities in general [2]. The search for this review only uncovered one review discussing studies on subjects with moderate or severe intellectual disability.

Verbal patients were most often studied with physiological measures for pain (375 combinations), followed by healthy adults and neonates (262 and 205, respectively). The physiological method most used to measure pain is the (f) MRI (193 combinations) followed by heart rate variability (184) and heart rate (169). Both (f) MRI and heart rate variability were most often measured for pain in verbal patients (in 193 studies) and healthy adults (in 141 studies), while heart rate was most often measured for pain detection in neonates (in 80 studies). Heart rate was also the most found method for measuring pain in children (in 32 studies) and nonverbal patients (in nineteen studies). With people with moderate or severe intellectual disability as a subject group, respiratory rate was measured for pain in four studies, while only one study with healthy adults as a subject group used respiratory rate to measure pain. The relatively new way to detect pain with automated facial recognition was used in four studies and only with verbal patients.

### 3.3. Pain Assessment per Method

Among the 540 studies discussed, different subject groups participated and the focus of studies also varied greatly. Care was made to look for similarities and material to compare among studies. Results per measurement method are displayed in Table 4. Table 4 shows that pupillometry, cerebral blood flow velocity (CBFV), and respiratory and muscular measures were among the low invasive measures that showed both the ability to measure pain and consistency in results among pain studies. Both pupillometry and CBFV have not yet been researched as a pain assessment method often but show promise in this category. The two respiratory measures and muscle tension all showed inconsistent or no results among one certain subject group, but it is unclear whether this is due to the measurement method or the study design.

Among the promising, yet more invasive pain assessment methods are magnetic resonance imaging (MRI), hormonal analyses, electromyogram, and genetic research. MRI and hormones can measure both acute and chronic pain responses, while genetics can only measure changes as a result of chronic pain and the electromyogram is only used for acute pain measurement. Few studies were found that used either hormonal analyses, electromyogram, or genetics as a pain measurement method, so this may limit the scope of these results. Electromyogram, MRI, and genetics were most often used to compare chronic pain patients to healthy adults, so results among other subject groups are based on few studies.

The remaining methods show inconsistent results among at least one subject group, which makes the results difficult to generalize, or show doubtful results as a pain measurement method. Skin conductance responses to pain were only consistently found in healthy adults, and the promising new technique of automatic facial recognition is still influenced by too many personal and environmental factors.

### 3.4. Technology Readiness Levels

Technology readiness levels indicate the technological maturity of an instrument. This can range from (1) only theoretical knowledge to (9) certified and used daily. Per modality shown in the taxonomy above one technological instrument was chosen, based on most use or most described in the articles in the taxonomy. Of course, the instrument had to be technical in order to determine a TRL. Heart and respiratory rates are often observed and computed manually by a nurse, but the determination of a TRL was based on a technical instrument that computes this automatically. Technology readiness levels per modality are reported in Table 5.

The average TRL of all (most used or most described) instruments together was eight, which is not surprising, considering that the instruments used in hospitals (such as an MRI-scanner or a fingertip pulse oximeter) are certified and used daily. The newest method, automated facial recognition, has the lowest TRL (four). The software has been validated in a lab, but not yet in a relevant environment with a patient group. The skin conductance measurement also has a moderate TRL (six). This is based on the fact that there has been a validation of a prototype in a hospital or clinic, but the demonstration of a GSR-measurement for pain in an operational way has not yet been done. There are certified instruments and software to use with GSR-electrodes, but these need to be adjusted to measure pain.

## 4. Summary of Evidence

In this systematic review, the physiological methods to assess pain in humans were made. From 29 reviews found, 540 articles described 1054 unique combinations of the physiological assessment method and subject group. When these combinations were presented in a graph, it showed a clear trend that the more invasive methods are most commonly used to assess pain in the least vulnerable groups. Moreover, in some of the vulnerable subject groups (those with mild or severe intellectual disability), there is hardly any research done on a physiological assessment method for pain. Since the review by De Knegt et al. in 2013, only two studies were conducted concerning physiological measurements of pain in persons with ID [38, 39]. Since people with severe intellectual disability are mostly unable to express their pain, and show different nonverbal signs of pain than expected (e.g., freezing of face and/or body), a consequence is that their caregivers do not easily recognize their pain. Therefore, there is a great need for research examining the precision and reliability of physiological assessment of pain in persons with severe intellectual disability.

This study shows that half of the most often used instruments reached the maximum level of technological maturity and another quarter of the instruments reached the penultimate level. Technology readiness of instruments used to measure physiological indications of pain is generally high. The only exception is the relatively new method of automated facial recognition software. Although facial recognition in general is making real strides, the automated recognition of emotions and facial expressions still needs to be developed further in order for it to be able to be used in the assessment of pain. Although when this technology reaches the point when it can be used accurately and reliably, there is still the question of whether it can be used on all subject groups, seeing as expressions of pain differ in persons with ID.

### 4.1. Limitations

For this study, a thorough and systematic search was made for published reviews between 2007 and 2019. Reviews that were primarily unavailable to the researchers and were searched for by addressing the authors and for hand selection reference lists of the included reviews were examined. However, there may have been eligible reviews that were not added such as unpublished reviews or those published before 2007. On the other hand, great efforts were made to include as many reviewed articles as possible by adding English, Spanish, Dutch, German, and French reviews. Therefore, the vast majority of review articles was examined.

The searches for articles included in the reviews could also result in articles missed, due to unavailability or language barriers. Even so, the amount of articles assessed (540) is deemed sufficient to give an overview of the trend in research and the results shown in the taxonomy. Therefore, this study gives an overview of research trends that should be quite similar to all research conducted.

Of the 29 reviews discussed in this review, only three used a systematic method and half assessed the quality and risk of bias in the included articles. When the risk of bias was assessed, it was generally scored as moderate or high by the authors of the reviews and the quality of evidence was usually moderate or low. Therefore, when studies selectively report their findings, this is copied by the reviews describing those studies and ultimately also in this review.

The taxonomy that shows the trend in physiological pain assessment methods and subject groups is based on the number of combinations of the method and group discussed per article. The taxonomy is not ordered according to the sizes of participant groups the methods are used on, but on the number of articles were that certain combination was discussed. The trend, therefore, might be different if the combinations were ordered on participant group sizes. Nonetheless, the way the taxonomy was ordered gives a clear overview of research trends in pain assessment. Otherwise, the trend would mostly show which participants are more easily reached by researchers, rather than which combinations of the method and subject group are more worthwhile for being studied.

## 5. Conclusions

Technology readiness of instruments used to assess pain is generally high. Some new and less used technologies, such as facial recognition and skin conductance still need to be developed further. In general, it can be said that instruments used in hospitals and clinics to assess pain in patients physiologically are technologically mature.

Whether all proposed methods of pain assessment can be used specifically to measure the existence of pain is another matter. In general, the examined studies show evidence that respiratory measures, muscle tension, MRI, CBFV, hormonal analysis, and pupillometry can reliably indicate acute pain, although not all of these methods were often studied, and if they were often on the same two subject groups (chronic pain patients and healthy adults). Cardiovascular methods were studied among many different subject groups but do not seem to give a clear indication that they respond to pain or nociception. There is little evidence also for electrodermal activity and near-infrared spectrometry as pain assessment methods. Brain scan techniques and genetics on the other hand seem to be useful in finding anomalies in chronic pain patients compared to healthy controls. The use of computer-based facial recognition software to detect pain needs to be further developed to be viable. In general, there is a need to find further evidence on the ways to measure pain physiologically, especially for those that are unable to communicate or express their pain. And specifically, there may be a task for (medical) engineers to be involved in instrument development and improvement.

The taxonomy shows a trend that the more invasive pain assessment methods are often only used on the less vulnerable subjects, while on the more vulnerable patients, less invasive pain assessment methods are used. As a result, this could suggest that researchers are hesitant to use more invasive pain assessment methods with subjects where informed consent is difficult to obtain. Very little research on physiological pain assessment is done with persons with intellectual disabilities. Participants with intellectual disability should generally be included more in scientific research, especially on subjects as pain, for pain relief will have a positive impact on their general wellbeing.

## Figures and Tables

**Table 1:** Inclusion and exclusion criteria.

Criteria	Inclusion	Exclusion
Population	(i) Human study	(i) Animal study
Instruments	(ii) At least one physiological measurement	(ii) No physiological measurement
Outcome	(iii) A clinical pain measure physiologically obtained	(iii) No clinical pain measure (iv) Only a self-report measure
Report	(iv) Review, systematic review, meta-analysis (v) Full-text available	(v) Article, letter to the editor (vi) Full-text not available
Period	(vi) 2007–2019	(vii) All other years
Language	(vii) Dutch, English, French, German, Spanish	(viii) All other languages

**Table 2:** Scoring for invasiveness of modalities and vulnerability of subject groups.

Invasiveness physiological modality			Vulnerability subject group		
Drastic	0	Not drastic or privacy invading	Communicative capability	0	Limited non-verbal and not verbal
	1	Mildly drastic		1	Limited verbal
	2	Moderately drastic		2	Both verbal and non-verbal
	3	Very drastic or privacy invading	Competence	0	No or very little competence
Duration	0	Up to 1 hour		1	Limited competence
	1	From 1 up to several hours		2	Normative competence
	2	From several hours up to 1 day	Illness	0	Illness, injury, or disability
	3	More than 1 day		1	No illness, injury, or disability

**Table 3:** Average scores for invasiveness of modalities (left side of the table) and vulnerability of subject groups (right side of the table).

Method	Average score	Subject group	Average score
Respiratory rate	0.00	Comatose patients	0.17
Respiratory analysis	0.33	People with severe ID	0.33
Heart rate	0.67	Neonates	1.83
Muscle tension	0.83	Children patients	3.00
Body temperature	1.00	People with moderate ID	3.50
Skin conductance	1.17	Verbal patients	3.83
Heart rate variability	1.33	Healthy adults	5.00
Blood pressure	1.50		
Pupillometry	1.67		
Cerebral BFV	1.83		
Facial recognition	2.00		
ECG	2.67		
EEG	3.00		
(f) MRI	3.83		
PET/SPECT	4.00		
Hormonal analysis	4.17		
Needle-based EMG	4.33		
Genetics	6.00		

**Table 4:** Pain assessment results per measurement method.

Method		Subjects	Able pain measure	Consistent across studies^*∗*^	Limitations
Electroencephalogram (EEG)		Neonates, infants, CPPs, ICU patients	Yes	Moderate	Influenced by opioids, not consistently found in neonates

Cardiovascular measures	Heart rate	Neonates, infants, TBI patients, ICU/OR patients, CPPs, people with SID, healthy adults	Doubtful	No	Variable results among brain injured patients, reduced reaction in CPPs, no reaction in SID
	Heart rate variability	Neonates, infants, ICU/OR patients, healthy adults	Yes	Moderate	Inconsistent among infants in the first year of life
	Body temperature	Neonates, infants, healthy adults	Yes	Moderate	Inconsistent among healthy adults
	Blood pressure	Neonates, infants, TBI patients, OR patients, healthy adults	Doubtful	No	Blood pressure responded inconsistently to pain

Respiratory measures	Respiratory rate	Neonates, infants, TBI patients, people with SID, healthy adults	Yes	Yes	Respiratory ‘irregularities' were not related to acute pain in persons with SID
	Respiratory analysis	Neonates, infants, TBI patients	Yes	Yes	Oxygen saturation was not favored for pain in neonates

Muscular measures	Muscle tension	Infants, ICU/OR patients, healthy adults	Yes	Yes	In healthy adults muscle tension response was only found with intense and prolonged pain
	Electromyogram	CPPs, healthy adults	Yes	Yes	Few studies

Electrodermal activity		TBI patients, OR patients, healthy adults	Doubtful	No	Only consistently found in healthy adults

Pupillometry		Infants, OR patients, CPPs, healthy adults	Yes	Yes	Few studies

Brain scan	MRI	CPPs, healthy adults	Yes	Yes	Different studies focused on different areas
	NIRS	Neonates, infants	No	Yes	Presence of pain on a cortical level was not found
	CBFV	Infants, CPPs, OR patients, people with SID	Yes	Yes	Few studies
	SPECT	Infants, CPPs	Yes	No	Activity varied greatly across studies

Hormonal analysis		Neonates, CPPs	Yes	Yes	Few studies

Genetics		CPPs, healthy adults	Yes	Yes	Not yet validated in large human cohorts

Automatic facial recognition		CPPs	Doubtful	Yes	Influenced by gender, age, ethnicity, movement, and lighting

*Note*. CPPs = chronic pain patients, ICU = intensive care unit, OR = operating room, TBI = traumatic brain injury, SID = severe intellectual disability, MRI = magnetic resonance imaging, NIRS = near-infrared spectroscopy, CBFL = cerebral blood flow velocity, PET = positron emission tomography, and SPECT = single-photon emission computer tomography. ^*∗*^ [2, 10–37].

**Table 5:** Technology readiness levels (TRLs) determined for one technological instrument (most used or most described) per detection method, ordered according to invasiveness.

Method	Instrument	TRL
Respiratory rate	Respiratory measure instrument	8
Respiratory analysis	Fingertip pulse oximeter	9
Heart rate	Visual stethoscope with oximeter probe	9
Muscle tension	Skin electrodes and EMG-monitor	8
Body temperature	Tympanic (ear) thermometer	9
Skin conductance	Skin electrodes, transmitter and display-software	6
Heart rate variability	Photo plethysmograph	7
Blood pressure	Digital sphygmomanometer	9
Pupillometry	Pupillometer	8
Cerebral BFV	Transcranial Doppler	9
Facial recognition	Facial recognition software	4
ECG	Skin electrodes and ECG-monitor	8
EEG	EEG cap and monitor	8
(f) MRI	MRI-scanner	9
PET/SPECT	PET-scanner	9
Hormonal analysis	Clinical centrifuge	9
Needle-based EMG	Needle electrode and monitor	7
Genetics	Genetic analysis instrument	9

## Data Availability

The data used to support the findings of this study are available from the corresponding author upon reasonable request.

## Supplementary Materials

Supplementary Materials
*Figure S1*. Flowchart for inclusion into the review. *Table S1*. Results of quality and transparency assessment for the 29 reviews (alphabetically). *Figure S2*. Technology readiness levels as described by the United States Department of Defense in accordance with instruments for physiological measurements of pain.
