# Supplementary material for: Physiological Measures of Acute and Chronic Pain within Different Subject Groups: A Systematic Review
Source: Pain Res Manag. 2020 Sep 3;2020:9249465. doi: 10.1155/2020/9249465 (PMC7487119; doi:10.1155/2020/9249465)
Supplement: Supplementary Materials — Figure S1. Flowchart for inclusion into the review. Table S1. Results of quality and transparency assessment for the 29 reviews (alphabetically). Figure S2. Technology readiness levels as described by the United States Department of Defense in accordance with instruments for physiological measurements of pain. [file 9249465.f1.docx]

**Supplementary Materials**


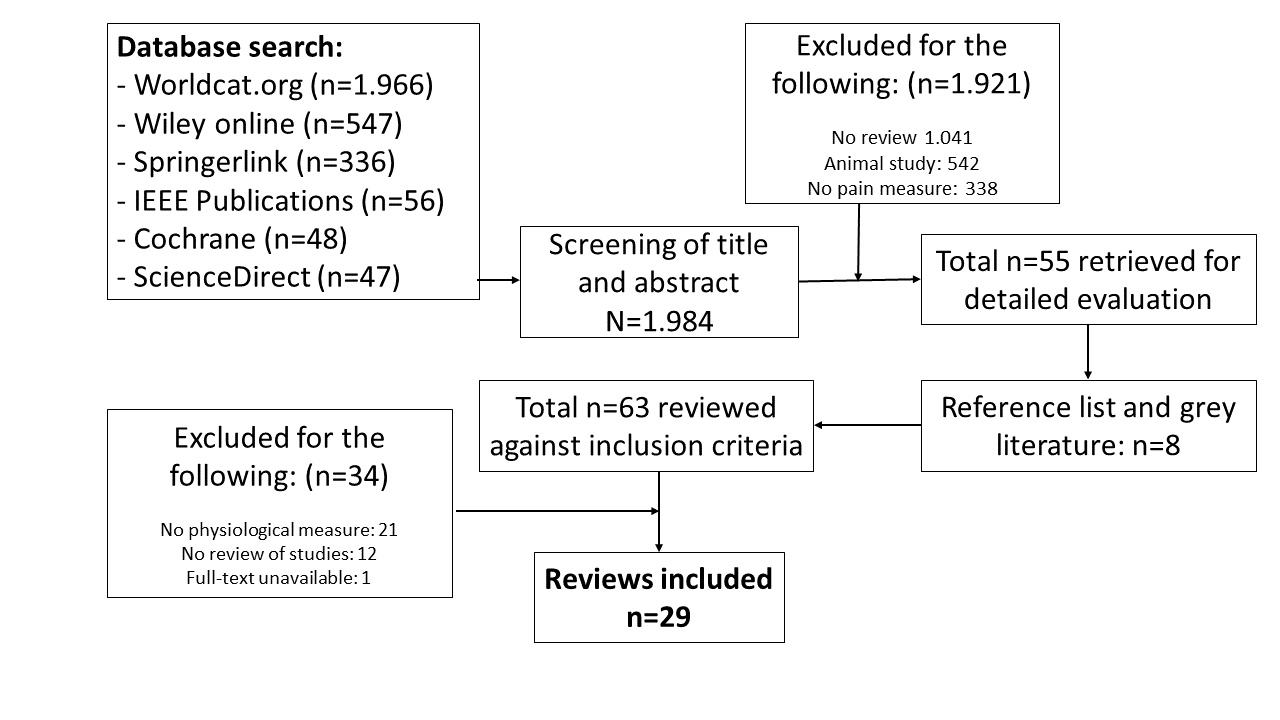


Figure S1. Flow chart for inclusion into the review.

Table S1. Results of quality and transparency assessment for the 29 reviews (alphabetically)

| Review | A) Validity | | | | | B) Precision | | C) Usefulness | | | Adequate answers | Systematic  guidelines |
| --- | --- | --- | --- | --- | --- | --- | --- | --- | --- | --- | --- | --- |
| (first) Author and year | 1 | 2 | 3 | 4 | 5 | 6 | 7 | 8 | 9 | 10 |  |  |
| Arbour, & Gélinas, 2014 | + | + | + | + | + | + | / | + | ? | + | 8/10 | no |
| Benoit, et al., 2017 | + | + | + | ? | + | + | - | ? | + | + | 7/10 | yes |
| Bentley, et al., 2016 | + | + | + | - | + | / | - | - | + | + | 6/10 | no |
| Bonan, et al., 2014 | + | + | ? | - | ? | + | - | ? | + | + | 5/10 | no |
| Cagnie, et al., 2014 | + | + | + | + | + | + | - | - | + | + | 8/10 | no |
| Choon Wyn Lim, et al., 2011 | + | + | + | + | + | + | + | ? | + | + | 9/10 | no |
| Coleman, et al., 2015 | + | + | + | - | + | + | / | - | ? | + | 6/10 | no |
| Coppetiers, et al., 2016 | + | + | + | + | + | + | / | - | ? | + | 7/10 | yes |
| De Jonckheere, et al., 2015 | + | + | ? | - | + | + | / | + | + | + | 7/10 | no |
| De Knegt, et al., 2013 | + | + | + | + | + | / | / | + | + | + | 8/10 | yes |
| Dorfman, et al., 2014 | + | + | + | + | + | + | / | + | + | + | 9/10 | no |
| Hatfield, & Ely, 2015 | + | + | + | + | + | + | + | ? | + | + | 9/10 | no |
| Heales, et al., 2016 | + | + | + | + | + | / | + | ? | + | + | 8/10 | no |
| Hecke, van, et al., 2015 | + | + | + | + | + | / | / | - | + | + | 7/10 | no |
| Henry, et al., 2011 | + | + | ? | - | + | / | - | ? | + | + | 5/10 | no |
| Koenig, et al., 2014 | + | + | + | - | + | + | - | + | + | + | 8/10 | yes |
| Koenig, et al., 2016 | + | + | + | - | + | + | + | + | + | + | 9/10 | yes |
| Kyle & McNeil, 2014 | + | ? | + | + | + | / | - | - | + | + | 6/10 | no |
| Manocha, & Taneja, 2016 | + | - | + | - | + | / | - | - | + | + | 5/10 | no |
| Meeus, et al., 2013 | + | + | + | + | - | + | / | ? | + | + | 7/10 | yes |
| Parker, et al., 2016 | + | + | + | + | + | + | + | ? | + | + | 9/10 | no |
| Pudas-Täkhä, et al., 2008 | + | + | ? | + | + | + | / | - | + | + | 7/10 | yes |
| Raeside, 2011 | + | + | + | + | + | + | - | - | + | + | 8/10 | no |
| Relland, et al., 2019 | + | + | + | + | + | + | + | ? | + | + | 9/10 | yes |
| Roulin, & Ramelet, 2012 | + | + | + | ? | + | / | / | ? | + | + | 6/10 | yes |
| Schmidt, & Martin, 2017 | + | + | + | - | - | / | - | - | + | + | 5/10 | no |
| Subramaniam, et al., 2018 | + | + | ? | ? | + | + | - | ? | - | ? | 4/10 | no |
| Waxman, et al., 2015 | + | + | + | + | + | + | / | ? | + | + | 8/10 | yes |
| Zamzmi, et al., 2018 | + | + | ? | - | + | + | + | - | + | + | 7/10 | no |

NB. + = adequate, - = inadequate, / = moderately adequate, ? = unclear

1) Did the review address a clearly focused question?

2) Did the authors look for the right type of papers?

3) Do you think all the important, relevant studies were included?

4) Did the review’s authors do enough to assess quality of the included studies?

5) If the results of the review have been combined, was it reasonable to do so?

6) What are the overall results of the review?

7) How precise are the results?

8) Can the results be applied to the local population?

9) Were all important outcomes considered?

10) Are the benefits worth the harms and costs?


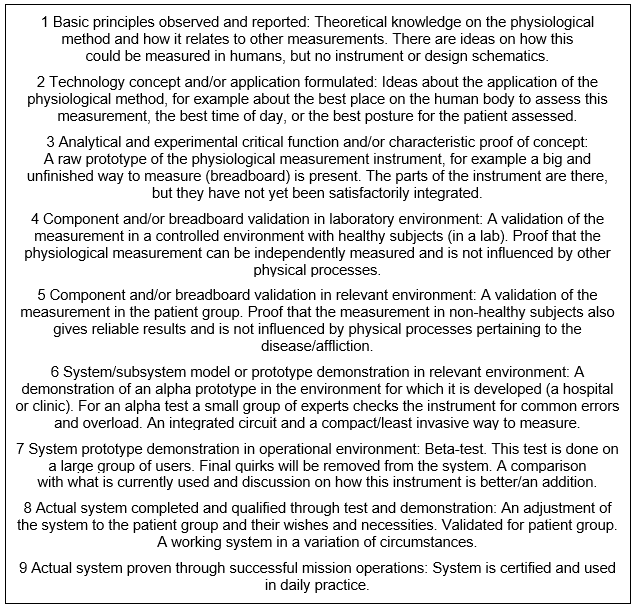


Figure S2. Technology Readiness Levels as described by the United States Department of Defense in accordance with instruments for physiological measurements of pain.
